# Antimicrobial Properties of Lyophilized Extracts of Olive Fruit, Pomegranate and Orange Peel Extracts against Foodborne Pathogenic and Spoilage Bacteria and Fungi In Vitro and in Food Matrices

**DOI:** 10.3390/molecules26227038

**Published:** 2021-11-21

**Authors:** Chrysanthi Mitsagga, Konstantinos Petrotos, Ioannis Giavasis

**Affiliations:** 1 Department of Food Science and Human Nutrition, School of Agricultural Sciences, Karditsa Campus, University of Thessaly, Terma Odou N. Temponera, 43100 Karditsa, Greece; cmitsanga@uth.gr; 2 Department of Agrotechnology, School of Agricultural Sciences, Geopolis Campus, University of Thessaly, Periferiaki Odos Larisas Trikalon, 41500 Larisa, Greece; petrotos@uth.gr

**Keywords:** olive fruit extract, pomegranate pulp extract, orange pulp extract, novel antimicrobials, natural preservatives, foodborne pathogens, spoilage microorganism, byproduct utilization

## Abstract

Several novel antimicrobials with different concentrations of olive, pomegranate, and orange fruit pulp extracts were produced from agricultural byproducts and, after lyophilization, their antimicrobial activity and potential synergistic effects were evaluated in vitro and in food samples against foodborne pathogenic and spoilage bacteria and fungi. The Minimum Inhibitory of the tested bacteria was 7.5% or 10%, while fungi were inhibited at a concentration of 10% or above. The optical density of bacterial and yeast cultures was reduced to a different extent with all tested antimicrobial powders, compared to a control without antimicrobials, and mycelium growth of fungi was also restricted with extracts containing at least 90% olive extract. In food samples with inoculated pathogens and spoilage bacteria and fungi, the 100% olive extract was most inhibitory against *E. coli*, *S. typhimurium,* and *L. monocytogenes* in fresh burger and cheese spread samples (by 0.6 to 1.8 log cfu/g), except that *S. typhimurium* was better inhibited by a 90% olive and 10% pomegranate extract in burgers. The latter extract was also the most effective in controlling the growth of inoculated fungi (*Aspergillus niger*, *Penicillium italicum*, *Rhodotorula mucilaginosa*) in both yogurt and tomato juice samples, where it reduced fungal growth by 1–2.2 log cfu/g at the end of storage period. The results demonstrate that these novel encapsulated extracts could serve as natural antimicrobials of wide spectrum, in order to replace synthetic preservatives in foods and cosmetics.

## 1. Introduction

Agroindustrial waste are posing a serious environmental and economic problem worldwide, since one third of the food produced for human consumption is disposed of as waste according to FAO, leading to environmental pollution, depletion of natural resources and compromised food security [1]. Therefore, UN countries have committed to reduce (by 50%) the food waste generated per capita at the retail and consumer level and decrease substantially the agro-industrial waste along the food supply chain by 2030, within the United Nations Sustainable Development Goal 12.3 [1]. For this reason, in the last decades the food industry has been seeking ways to utilize agricultural waste in order to tackle waste management and also produce novel products of added value. Notably, the utilized food byproducts, especially fruit and vegetable by-products, often consist of valuable components [2] such as phenols, flavonoids, pigments, and organic acids, which can be used in food and feed as natural antioxidants and/or antimicrobials [3,4,5], or in phytoprotection [5,6,7] and in cosmetics and pharmaceuticals [8], potentially replacing synthetic chemical preservatives. Our research has focused on the utilization of olive mill waste, orange peel, and pomegranate peel waste and their potential application as natural antimicrobial substances in vitro against important food pathogens and spoilage microorganisms and in the formulation of natural substances that can replace chemical additives in food matrices, in accordance with consumers’ demand for “green label” foods. Olive mill wastewater is the liquid by-product of the olive oil production industry. According to Chowdhury et al. [9], olive mill waste water (OMW) has a high organic load (BOD 89–100 g L^−1^, COD 80–200 g L^−1^), which is characterized by high phytotoxicity. The dark color of OMW is mainly caused by the abundant phenolic compounds, which are also responsible for the phytotoxicity [10]. However, these phenolic compounds, or polyphenols of OMW, also have strong antioxidant and antimicrobial properties, especially after encapsulation of the olive extracts [11,12].

Orange fruit pulp and peels represent a large amount of the total worldwide production of >73 million tons of orange fruits [5] and their disposal is problematic especially in the orange juice industry. Interestingly, these byproducts of juice production are rich in phenols, flavonoids, flavone glycosides, organic acids, as well essential oils, which are reported to exert antimicrobial activity against several bacterial pathogens [13,14,15].

Pomegranate waste (peel and seeds) are produced in a similar manner during the processing of pomegranates (*Punica granatum* L.) for the production of juice. Pomegranate fruit byproducts and extracts thereof have high concentrations of polyphenols and tannins, thus demonstrating strong antioxidant and antimicrobial activity against several pathogens and spoilage microorganisms, which depends partly on the extraction method and the part of the fruit that is used [16,17,18,19]. In fact, peels appear to be richer in phenols and antioxidant compounds compared to pomegranate pulp [17].

After appropriate membrane filtration, purification, and encapsulation of plant extracts without use of chemical solvents (i.e., consecutive dialfitration with the addition of water, ultrafiltration to remove high molecular weight solids and reverse osmosis to remove salts, followed by encapsulation in maltodextrin via freeze drying), a polyphenol-rich extract can be obtained from OMW [20], which has been effective in controlling the growth of several fungal and bacterial pathogens [5,12], as well as enhancing the growth of beneficial lactic acid bacteria when used at low concentrations [21]. Using an adequate concentration of these natural extracts is crucial in exhibiting inhibitory effects against microorganisms, but their dark color and intense taste can be deterrent in food applications; this can be alleviated via encapsulation in edible carriers such as maltodextrin [21].

In the present study, polyphenol-rich extracts of OMW, orange and pomegranate peel were combined in different ratios and encapsulated into lyophilized powders, with the scope of utilizing simultaneously the main byproducts of three large agricultural industries and also enhancing the already documented antimicrobial activity of encapsulated olive polyphenols [5,12], by a potential synergistic affect with pomegranate and/or orange pulp extracts, which can also be rich phenols and flavonoids, especially after appropriate optimization of extraction conditions [22,23].

## 2. Results

### 2.1. Measurement of the Total Phenols, Flavonoids and pH of Selected Lyophilized Antimicrobial Powders

The coding of all antimicrobial extracts and the composition of each encapsulated extract are explained in detail in Section 4.1 (Materials and Methods).

The measurements of phenols and flavonoids concentration of the selected antimicrobial powders are shown in Table 1. Extract 100/0/0 was richest in phenols (47.86 g/Kg) expressed as equivalents of gallic acid, while extract 80/20/0 had the highest content of flavonoids, expressed as equivalents of quercetin. All extracts had a slightly acidic pH around 5, among which the extract 80/0/20 had the lowest pH.

It seems that a very high content of olive extract (100%) contributes to a higher concentration of phenols, but a relatively modest content of flavonoids, while a high ratio of pomegranate extract (20%) leads to a high content of flavonoids, but also contributes to the phenol concentration to some extent. A relatively high content (20%) of orange extract may reduce the pH of the extract, but results in low phenol content and is not very rich in flavonoids, either.

### 2.2. Minimum Inhibitory Concentration (MIC) and Minimum Lethal Concentration (MLC)

Table 2 depicts the Minimum Inhibitory Concentration (MIC) of different cultures of foodborne pathogenic bacteria and pathogenic or spoilage fungi.

*E. coli* was more vulnerable to powder 90/10/0 for which MIC was 7.5%, while for all other powders MIC was 10%. For *S. aureus* the most effective powders with a MIC of 7.5% were 90/5/5, 90/10/0, 80/10/10, 80/20/0. *L. monocytogenes*, *S. typhimurium,* and *B. cereus* were equally susceptible to all powders which had a MIC of 7.5%, while *C. jejuni* was most resistant to powders 90/0/10 and 80/0/20 (which contained 10% and 20% ratio of orange extract, respectively). *C. perfringens* was equally resistant to all powders which had a MIC of 10%. With regard to the fungi, all tested cultures had a MIC above 10%, except for *F. oxysporum* which was most susceptible to powders 100/0/0, 90/5/5, and 80/10/10 (with a MIC of 7.5%), while all other powders had a MIC of 10% for *F. oxysporum*. Minimum lethal concentration (MLC) was higher than 10% for all bacterial and fungal cultures (data not shown), indicating that a higher concentration than 10% is needed for killing microbial cells with any of the tested powders, although a 7.5% or 10% could effectively control the growth of most bacteria and a few fungi (as shown by the MIC values).

### 2.3. Inhibition of Growth Measured by Optical Density

**Figure 1:**
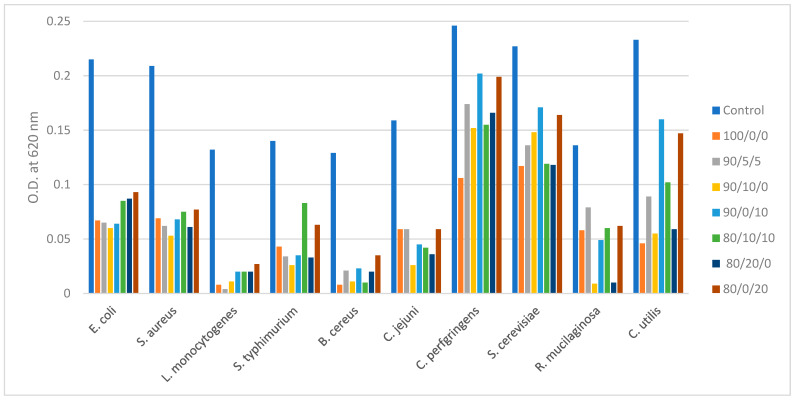
Measurement of optical density (O.D.) of liquid cultures of bacteria and yeasts in the presence of 7.5% of selected antimicrobial powders.

Since a 7.5% concentration was the minimum effective concentration of antimicrobial powders needed to inhibit the growth of many bacterial pathogens, this concentration was also applied in liquid cultures of bacteria and yeasts, where the optical density was measured, with or without the addition of antimicrobials, after appropriate incubation. The results shown in Figure 1 indicate that all tested powders conferred some antimicrobial activity in terms of reducing the optical density (O.D.) as well as the biomass of growing cells of all microorganisms. The largest reductions of O.D. were observed among bacterial cultures, compared to fungi (yeasts), which seemed more resistant, in agreement with the results of the MIC method. Powders 90/0/10 and 80/0/20 were more effective than the rest against *E. coli* and *S. aureus*, while 90/10/0 was the most inhibitory towards *S. typhimurium* and especially *C. jejuni.* Samples 100/0/0 and 90/5/5 were very effective in controlling the growth of *L. monocytogenes*, while 10/0/0 and 90/10/0 were also effective against *B. cereus* and *C. perfingens.* The powder that contained only olive extract (100/0/0) was also the most inhibitory in the case of *S. cerevisiae* and *C. utilis*, while *R. mucilaginosa* was better inhibited when the olive extract was combined with some pomegranate extract, as in the case of powders 90/10/0 and 80/20/0.

### 2.4. Mycelium Growth Assay

**Figure 2:**
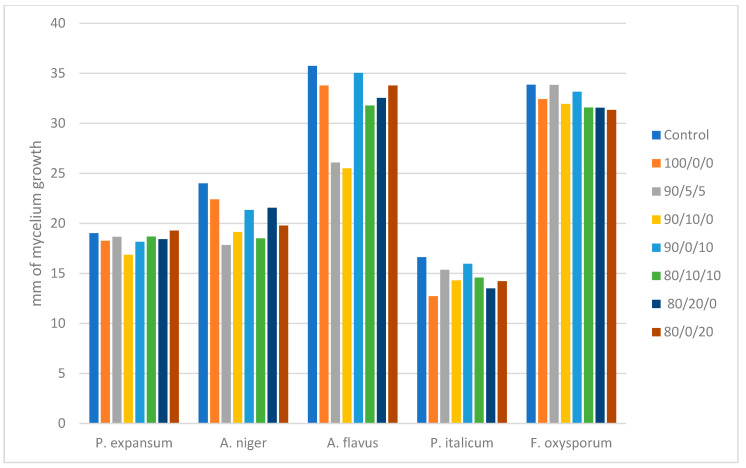
Mycelium growth of five different fungi with 10% of antimicrobial powders after 2, 4, and 7 days of incubation.

The mycelium growth assay of molds grown in the presence of 10% of antimicrobial powders revealed that the tested molds were less affected by the encapsulated antimicrobial extracts, in comparison to bacteria and yeasts. In fact, for some of the powders and the tested fungi, no practical restriction of mycelium expansion on agar plates was observed. As shown in Figure 2, *P. expansum* can be inhibited only slightly by powder 90/10/0, *A. niger,* can be inhibited mostly by powders 90/5/5 and 90/10/0. The powder 90/0/10, *A. flavus,* is affected mostly by 90/10/0 or 100/0/0. *P. italicum* is inhibited mostly by 100/0/0, while little antifungal effect is observed against *F. oxysporum* with any of the tested antimicrobials.

### 2.5. Antimicrobials Effects In Vivo (in Food Samples)

**Figure 3:**
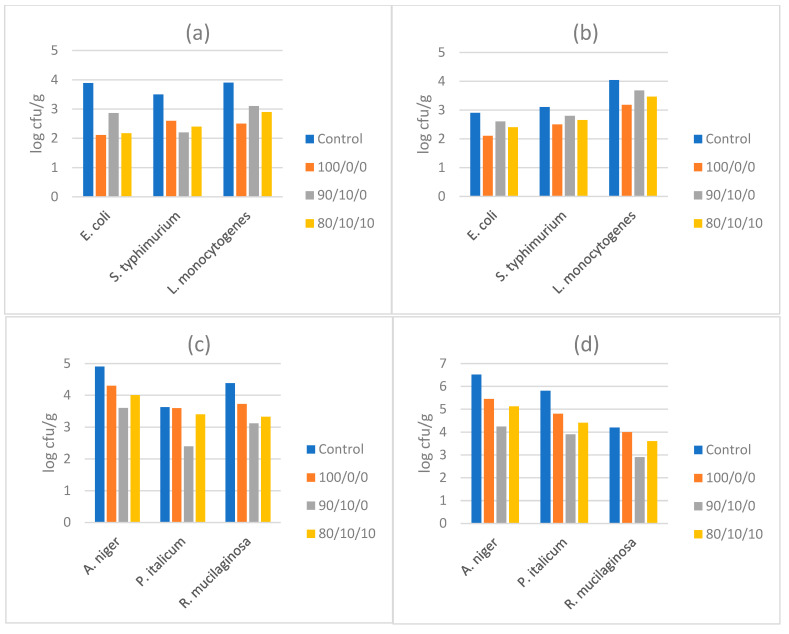
Populations (log cfu/g) of inoculated bacteria and fungi in four different food matrices, in the presence of selected antimicrobial powders, after appropriate storage. (**a**) Fresh burger after 4 days at 4 °C, (**b**) fresh cheese spread after 21 days at 4 °C (**c**) yogurt after 21 days at 4 °C, (**d**) tomato juice after 21 days at 4 °C. (Mean values of triplicate measurements are presented).

Based on the results of in vitro antimicrobial activity, three antimicrobial powders were selected for further tests in different types of food in order to serve as natural preservatives and help extend the shelf-life and control the growth of food pathogens in vivo. Namely, powders 100/0/0 and 90/10/0, which seemed to be the two best performing antimicrobials in vitro, as well as powder 80/10/10, which had a moderate antimicrobial effect in vitro and contained a ratio of 10% orange extract, were added in (a) fresh beef burger stored for 4 days at 4 °C, (b) fresh soft cheese spread stored for 21 days at 4 °C (c) yogurt dessert stored for 21 days at 4 °C, and (d) pasteurized tomato stored for 21 days at 4 °C. *E. coli*, *S. typhimurium,* and *L. monocytogenes* were inoculated and used as hygiene/safety markers in fresh burger and fresh cheese spread, while *A. niger*, *P. italicum,* and *R. mucilaginosa* were inoculated in yogurt dessert and tomato juice, where they served as spoilage indicators. The results of microbial populations after appropriate storage showed that powder 100/0/0 was the most effective in reducing the growth of *E. coli* and *L. monocytogenes* in both burger and cheese spread by 0.6 to 1.8 log cfu/g, although *S. typhimurium* was better inhibited by 90/10/0 in the burger and 100/0/0 in the cheese spread (Figure 3). With regard to the food matrices inoculated with fungi, the powder 90/10/0 was the most effective against all three fungal species, in both yogurt dessert and tomato juice, where it reduced fungal growth by 1–2.2 log cfu/g. This was a significant inhibition of microbial growth which could extend the shelf-life of these products, since fungi are the main source of spoilage in these products. Notably, the mixture of the three extracts in powder 80/10/10 worked better against fungi (compared to bacteria) in foods, and it seems that the presence of some orange extract (10%) along with some pomegranate extract (10%) could perform better as an antifungal in foods, in comparison to the previous in vitro tests.

**Figure 4:**
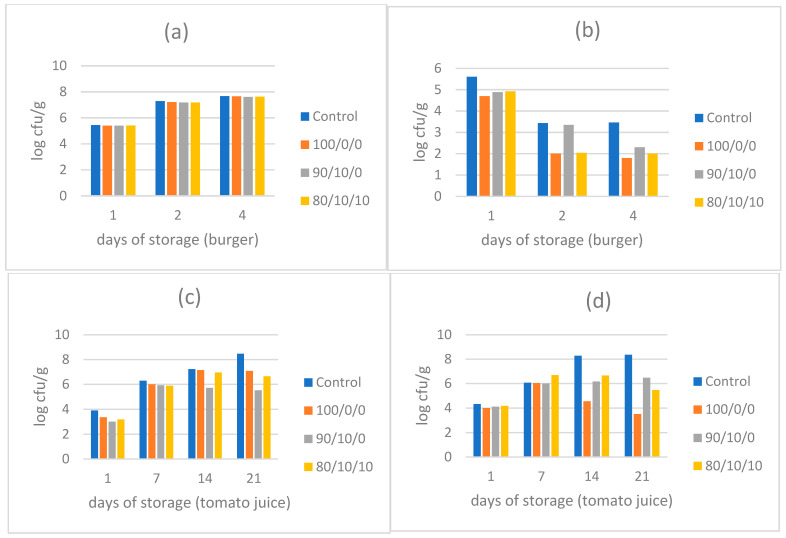
Populations (log cfu/g) of indigenous bacteria and fungi in two different food matrices, in the presence of selected antimicrobial powders, after appropriate storage at 4 °C. (**a**) Total Plate Count in fresh burger, (**b**) *Enterobacteriaceae* in fresh burger, (**c**) yeasts and molds in tomato juice, (**d**) lactic acid bacteria in tomato juice.

The same selected antimicrobial powders of encapsulated extracts were also tested for the efficacy against spoilage indicators of the indigenous microbiota of fresh burgers and pasteurized tomato juice, stored at 4 °C in open containers. The results (Figure 4) showed that Total Plate Count (TPC) was not affected by the presence of any of the antimicrobial powders at 0.5% concentration, at least within the first 4 days of refrigerated storage. However, this may be due to the fact that the antimicrobial powders were not sterile and carried their own indigenous microbiota, which could contaminate the sample and contribute to the population of Total Plate Count (TPC), since the product was not pasteurized/sterilized or cooked. On the other hand, *Enterobacteriaceae* were clearly affected by the presence of all extracts and especially powder 100/0/0 and 80/10/10, which could lower the population by up to 1.5 log. In pasteurized tomato juice, yeasts and molds were reduced mostly by powder 90/10/0 (by 1.5 log on 21th day of storage), while lactic acid bacteria (LAB) were inhibited by all powders at 0.5% concentration, from which the powder 100/0/0 seemed to be the most efficient, as it reduced microbial growth by ~ 2–2.5 log on the 14th and 21st day of storage.

## 3. Discussion

### 3.1. In Vitro Antimicrobial Activity

The antimicrobial efficiency of the different encapsulated antimicrobial powders was dependent on the tested microorganisms and the composition of each extract. The most sensitive bacteria according to MIC (Table 2) were *S. typhimurium*, *L. monocytogenes,* and *B. cereus* with a MIC of 7.5% for all tested extracts, while spore-forming *C. perfringens* was the most resistant bacterium with a MIC of 10% for all extracts. Comparatively, powder 90/10/0, which had the second highest content of both phenols and flavonoids (Table 1), also had the broadest antibacterial spectrum at a 7.5% concentration, as it could inhibit all tested bacteria, apart from *C. perfingens*, (which was inhibited only at 10% concentration). The only powder that could not inhibit *S. aureus* at 10% was the 80/0/20 (with the lowest content of phenols and 2nd lowest content of flavonoids).

The measurements of optical density (O.D.) of liquid cultures of bacteria and yeasts (Figure 2) showed that overall, the powders 100/0/0 and 90/10/0 had the highest antimicrobial activity against bacteria and yeasts, while the samples that were relatively rich in orange pulp extract (80/0/20 and 90/0/10) were the least effective, especially against fungi. Powder 80/20/0 (the one with the highest content of pomegranate extract) was also an effective antimicrobial, especially against yeasts.

In similar studies of pomegranate peel and olive leaf extracts (water, ethanol, or methanol extracts) [24] a MIC of 2.5% to 30% were obtained against different bacterial pathogens, where each tested pathogen had a different degree of sensitivity to the tested extracts. This is partly in agreement with our results, although we had followed a different procedure for producing the pomegranate pulp and olive fruit extracts (without the use of organic solvents). In the work of Seddiek et al. [24] the pomegranate peel extract had a better antibacterial activity compared to the olive leaf extract, however, in our study where the whole pomegranate pulp (peel, flesh, and seeds) and olive fruit pulp extract were used, it appeared that a high olive fruit extract, with or without some addition of pomegranate extract are optimal for antimicrobial activity. Obviously, the type, and composition of raw materials, or even the genotype/variety of the plant source used for each extract [25], as well as the extraction method determine the level of the antimicrobial effect. A MIC of about 10% or lower seems to be average for antibacterial activity, as in the case of extracts of pomegranate arils, which had a MIC of 3–9% against different pathogenic bacteria and yeast in vitro [25].

With regard to antifungal activity, the presence of orange extract at 10 or 20% (powders 90/0/10 and 80/0/20) seemed to lessen the inhibitory effects (Figure 1), most likely due to the lower content of phenols and flavonoids in that extract. This is in agreement with the previous reports on the limited antifungal effects of orange pulp extracts, in contrast to the presence of some antibacterial activity of orange pulp methanol extracts in vitro [26]. In general, fungi and especially molds were more resistant to all tested extracts, compared to bacteria, and the measurement of mycelium growth (Figure 2) revealed that little inhibition should be expected for some molds (such as *P. expansum* and *F. oxysporum*) with any of the tested extracts. Comparatively, the most inhibitory effect against molds in vitro was obtained with powder 90/10/0 and to a lesser extent powder 100/0/0 (Figure 2), indicating a potential synergy of pomegranate and olive extract against some fungi. However, if the pomegranate extract ratio was increased to 20% (in powder 80/20/0) the inhibition of mycelium expansion could be impaired, which implies that olive extract is the most important antimicrobial agent in these powders and that if the olive extract ratio falls below 90%, the synergism with pomegranate may be lost.

In a previous study by Leontopoulos et al. [5] where similar extracts of olive and pomegranate pulp were tested against phytopathogenic molds, it was found that the 100% olive pulp extract had the optimal antifungal activity against most plant pathogens, although a 100% pomegranate was also very effective against some other molds. In the same study, a very good antifungal activity in vitro was usually observed when the major component (70–100% ratio) was an olive fruit extract and/or when pomegranate extract was added to at least 10% ratio. Orange pulp extract was the least effective antifungal agent in that study, in agreement with the results of the present study.

### 3.2. Antimicrobial Activity In Vivo (in Food Samples)

After the in vitro screening of antimicrobial capacity of the encapsulated extracts, their application in different food matrices was important in order to validate their efficiency in a more realistic and complex environment and estimate the practical benefits for food preservation and safety. In relation to the inoculated bacterial pathogens, the pure (100%) olive pulp extract was the most preferable for controlling bacterial growth in fresh burger and cheese spread (Figure 3). No (significant) synergism was observed between olive and pomegranate extract (powder 90/10/0), or olive, pomegranate and orange extract (80/10/10) and the reduction of the ratio of olive extract seemed to impair the antibacterial effect. This may be due to the fact that bacteria are more vulnerable to the high phenol content and most importantly the composition of phenols of olive fruit which are present in powder 10/0/0.

In contrast, the inoculated fungi in yogurt and tomato juice were better inhibited with a combined extract of olive with some pomegranate, or even some orange pulp (Figure 3). The powder 90/10/0 had the highest antifungal effect against the three fungi present in yogurt and tomato juice, while powder 80/10/10 also worked better than a pure olive fruit extract. This may mean that fungi are more susceptible to a higher content of flavonoids resulting from the presence of pomegranate, or a broader variety of phenols (such as punicalagin) with flavonoids (such as kaempferol, quercetin, myricetin, luteolin, and apigenin), tannins (such as ellagitannin) and anthocyanins (such as pelargonidin), which are present in pomegranate peel and/or pulp and associated with antimicrobial activity [27,28,29].

In non-inoculated food samples (Figure 4), the above results of inoculated samples were verified to a great extent. *Enterobacteriaceae* in fresh burgers and LAB in pasteurized tomato juice were better controlled with powder 100/0/0, meaning that a 100% olive fruit extract having the maximal content of phenols was the most effective. Similarly, lactic acid bacteria in tomato juice were mostly inhibited by the same powder, which seems to be optimal for antibacterial activity. Nevertheless, TPC did not seem to be affected by this or any other antimicrobial powder, at least in the first four days of storage of fresh burgers. This was most likely due to the fact that the powders were not sterile and the product was not cooked or thermally treated to kill any contaminating microbiota of encapsulated powders, thus the potential inhibitory effect of the encapsulated extracts was most likely counter-balanced and neutralized by the increase of TPC caused by the natural microbiota of each powder. In the case of yeast and molds in tomato juice, the powder 90/10/0 containing 90% olive extract and 10% pomegranate extract was clearly the most effective antifungal agent in this food, showing a potential synergism of pomegranate extract with olive extract against fungi, which was also observed in inoculated samples (Figure 3). It seems that the high content of flavonoids in powder 90/10/0, along with the combined phenol content of olive and pomegranate extract is preferable for controlling fungal spoilage in food samples.

The antimicrobial and potential preservative effect of pomegranate extracts was also recently investigated, in combination with avocado extracts in several food samples. It appeared that pomegranate pulp extracts had notable antifungal activity, but less pronounced antibacterial activity in perishable food products, most likely due to their high phenol and tannin-rich content [30]. Pomegranate extracts have been successfully used as antifungal agents for the preservation of fruits [31,32]. In addition, their antibacterial and antioxidant activity has been exploited in muscle foods such as meatballs, meat pate΄, chicken chili and chicken lollipops, several fish products, as well as dairy and fruit or vegetable products, where the water or alcohol extracts of pomegranate could inhibit the growth of pathogens such as *L. monocytogenes*, *Bacillus subtilis*, *Bacillus cereus*, Escherichia coli, *Staphylococcus aureus,* and fecal coliforms [33,34,35,36,37]. In processed poultry products the addition of pomegranate extracts could reduce TPC by 1.5–2.0 log and thus extend the shelf life [34], although the antimicrobial protection in foods related to storage temperature is reduced at temperatures above 4 °C [35].

As observed previously, the extracts of olive fruit that are disposed of as olive mill waste are rich in a variety of polyphenols and are efficient as antimicrobials against many phytopathogenic fungi and could be applied in the biological protection of fruits and vegetables from spoilage molds [5,6,38,39]. Besides, a significant antibacterial activity has also been reported for olive leaf extracts or olive fruit pulp extracts, which have been used in the preservation of minced meat, sausages, meat cubes, or seafood [40,41], due to their activity against food pathogens and spoilage bacteria.

The effectiveness of the olive fruit pulp or leaf extracts has already been exploited in commercial products for use in food, such as Medoliva^®^, Medoliva Plus^®^ [42] and HIDROX^®^ [43], which is especially rich in hydroxytorysol. However, one should keep in mind that in order to inhibit microbial growth, a combination of multiple polyphenols and antimicrobial polysaccharides (i.e., multiple hurdles) found in these olive extracts is more effective than the single use of pure and single active substances such as hydroxytyrosol, quercetin, or oleuropeine [44,45,46]. This highlights the complex interactions that exist in the mechanisms of antimicrobial activity in such plant extracts [40].

Apparently, as in all functional plant extracts, the composition and effectiveness of every extract varies according to the extraction conditions and the solvents used [16,33,36], as in the case of methanol extracts of pomegranate peel, which are richer in phenolsand flavonoids than the water extracts [16]. However, using an organic solvent is a much more expensive and less sustainable solution, and in this context, one of the major advantages of the present study was that only water extracts were used, which are also readily compatible with all food and cosmetics applications.

Overall, based on the results of the present research, it can be deduced from the above that the water extracts of olive, pomegranate, and orange pulp can be utilized as effective antimicrobials in food or other applications, such as cosmetics, where they could serve not only as antimicrobials, but also as antioxidants. After a thorough screening of optimal powders via in vitro tests, the use of the relatively optimal antimicrobial powders in foods showed that the composition affects the antimicrobial activity: a single use of olive extracts seemed to be preferable for antibacterial activity in foods, while a combined use of olive extract (as the major ingredient) with some pomegranate extract (at 10% ratio) results in maximal antifungal activity, possibly due to synergistic effects and the presence of a broader spectrum of antimicrobials from two different plant sources.

The goal of this work was to demonstrate the antimicrobial efficacy of these novel encapsulated natural ingredients, and it was found that olive fruit extracts, with or without the addition of some pomegranate extracts offer the optimal antimicrobial activity and are potential candidates for the replacement of synthetic preservatives in foods and cosmetics, or even antibiotics in animal breeding, which would be worth further investigation in the future.

## 4. Materials and Methods

### 4.1. Preparation of Novel Olive, Pomegranate and Orange Extracts and Lyophilized Powders Thereof

Three types of extracts were used. The olive fruit extract was produced commercially and obtained from the company Polyhealth S.A. (Larisa, Greece) in liquid form by the name Medoliva Liquid, which is an aqueous extract of *Olea Europea* (olive) fruit isolated after dialfiltration, microfiltration, and reverse osmosis of olive mill waste (OMW) via a patented process, and contains several olive phenols, mainly hydroxytyrosol, tyrosol, caffeic and coumaric acid, catechins, and anthocyanins [42]. The other two aqueous extracts were derived from the *Punica granatum* (pomegranate) juice pomace and from *Citrus sinensis* (L.) (orange) juice pomace, which are the solid by-products of the pomegranate and orange juice industry, respectively. Before obtaining the pomegranate and orange pomace aqueous extracts by “green” vacuum microwave extraction, the two extraction processes were optimized by a novel method, which has been described previously [22,23]. The two initial optimized extracts from pomegranate and orange pomace were treated with reverse osmosis at ambient conditions (temp 25 °C) under pressure of 26 bars, in order to achieve a 5-fold concentration of each extract.

**Figure 5:**
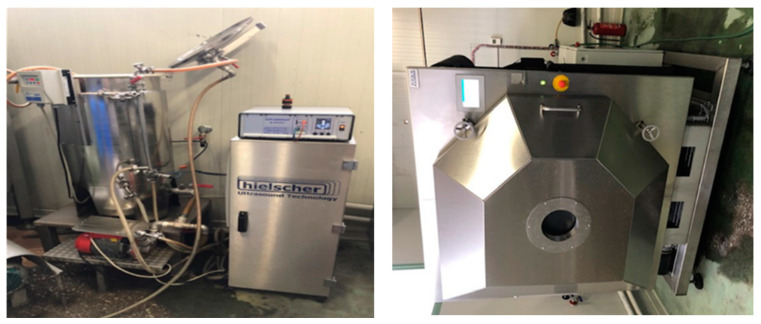
Industrial ultrasound homogenizer, Hielscher model UIP1000hdT (**left**) and industrial freeze dryer Zirbus GmbH model EKS 100-109 (**right**).

For the production of encapsulated powders of the above aqueous extracts, the three extracts were mixed in various ratios (Table 1) and in each mixture maltodextrin DE18 dry powder (120 g) and distilled water was added, up to a final mass of 600 g. Then this mixture was homogenized by an Ultrasound homogenizer (model UIP1000hdT, 1000 W, 20 kHz, Hielscher, Teltow, Germany) (Figure 5) and the finished liquid, which had a dry solid content of about 30% *w*/*w*, was finally freeze dried using an industrial freeze dryer with a total capacity 100 Kg (model EKS 100-10, Zirbus Technology GmbH, Bad Grund, Harz, Germany) (Figure 4). The total lyophilization cycle lasted 21 h, including 2 h of freezing at −35 °C.

Different antimicrobial powders were prepared, based on encapsulated olive, pomegranate, and orange extracts, in order to determine their potential synergistic action in relation to their antimicrobial activity. The initial number of the lyophilized powders with different concentrations of olive, pomegranate, and orange extracts were 72, as described previously [5]. These were limited to the seven best powders shown in Table 1, based on preliminary in vitro tests (data not shown) and previous research [5] which showed that for optimal antimicrobial activity olive extract was the most crucial ingredient and antimicrobial effects were reduced if the olive extract was used at a ratio below 70%. Therefore, the encapsulated antimicrobial powders studied here were produced from a liquid extract composed of at least 80% olive fruit extract and 0–20% of pomegranate and/or orange extract, in order to study any potential synergistic effects of the three extracts (when olive extract was the major component). The coding of samples of encapsulated extracts is also explained in Table 3.

### 4.2. Measurement of Total Phenols and Flavonoids of the Lyophilized Powders

For the measurement of the total phenolic content (expressed as gallic acid) a modified Folin-Ciocalteu method was used [47]. According to this method, each encapsulated antimicrobial powder (sample) was appropriately diluted (e.g., 1/10 dilution ratio) in deionized water and 0.2 mL of this solution was mixed with 10.8 mL of distilled water, 8 mL of Na_2_CO_3_ solution (prepared by dissolving 75 g Na_2_CO_3_ in 1 L distilled water), and 1 mL Folin-Ciocalteu reagent. All samples were homogenized in the vortex and then placed in a dark cabinet for 1 h at room temperature. After this incubation, the absorbance was measured at 750 nm using a UV-Vis spectrophotometer (DR 5000, Hach Lange, Loveland, CO, USA). Deionized water was used as a blank sample. The standard curve was prepared with standard solutions of gallic acid, with concentrations ranging from 0 to 1000 µg/mL.

Total flavonoids were measured by the aluminum chloride method described by Hassan et al. [48], with a few modifications. Briefly, 1 g of antimicrobial powder was dissolved in 80% ethanol up to a total volume of 25 mL. After mixing in the vortex, it was left overnight at room temperature and then centrifuged at 5000 rpm for 10 min. The supernatant was filter with plain filter paper and 0.5 mL of this sample was mixed with 1.5 mL of 95% Ethanol, 0.1 mL of 10% AlCl_3_, 0.1 mL of 1 M Potassium Acetate, 2.8 mL distilled H_2_O and incubated at room temperature for 30 min, after which the absorbance was measured at 415 nm. A blank sample was prepared by replacing AlCl_3_ with 0.1 mL H_2_O distilled. The standard curve was prepared with quercetin standard solutions (in 89% ethanol), ranging from 0 to 100 µg/mL concentration.

### 4.3. Cultures of Microorganism

The in vitro antimicrobial assessment of these novel powders was evaluated against several foodborne pathogens and spoilage microorganisms, which are particularly important for food safety and quality. The bacterial and yeasts cultures used in this study were obtained by DSMZ Culture Collection (Braunschweig, Germany), while the molds were kindly provided by the Benakion Phytopathological Institute of Greece.The bacterial cultures were maintained in Tryptone Soy Broth (TSB) (Neogen, Lansing, MI, USA), while fungal cultures were maintained in Potato Dextrose Broth (PDB) (Neogen, Lansing, MI, USA). For the determination of the population in food matrices, each target microorganism was cultured in (selective) agar medium. *Escherichia coli* was cultivated in TBX agar (Oxoid, Basingstoke, UK) at 37 °C for 24 h, *Enterobacteriaceae* were counted in Violet Red Bile Glucose agar (Neogen, USA) at 37 °C for 24 h, *Listeria monocytogenes* was counted on Harlequin Listeria Chromogenic Agar (Ottaviani and Agosti) (Neogen, Lansing, MI, USA), supplemented with Brilliance Listeria Differential supplement and OCLA (ISO) Selective supplement, *Salmonella typhimurium* was cultivated on XLD agar (Oxoid, Basingstoke, UK) at 37 °C for 24 h. Lactic acid bacteria were cultivated in MRS agar (Oxoid, Basingstoke, UK) at 37 °C for 48 h and *Clostridium perfringens* was counted in TSC agar (Oxoid, Basingstoke, UK) at 37 °C for 48 h under anaerobic conditions. With regard to the enumeration of fungi, all yeast and molds were cultivated in Potato Dextrose agar (Oxoid, Basingstoke, UK) at 25 °C for 3–5 days.

### 4.4. Minimum Inhibitory Concentration (MIC) and Minimum Lethal Concentration (MLC)

In this method, the tested antimicrobial powders were added in test tubes at a concentration of 0% (control without antimicrobial powder), 2.5%, 5%, 7.5%, and 10% in 10 mL of either TSB (for bacteria) or PDB (for fungi). After sterilization and inoculation with 0.1 mL of a fresh bacterial or fungal culture, the bacterial cultures were incubated for up to 48 h and fungal cultures were incubated for up to 96 h. After the incubation, the presence or absence of growth (evidenced by sediment or turbidity for bacteria and yeasts, and the appearance of superficial mycelium on top of the liquid culture for molds) was recorded. The minimum concentration of antimicrobial powder where no growth was observed was recorded as MIC. For the determination of MLC, all tubes at or above the MIC level were sub-cultured in fresh TSB/PDB without the addition of antimicrobials, and after appropriate incubation the test tubes were again checked for evidence or absence of growth. Absence of growth at that second stage meant that cells were dead at the corresponding concentration of the first step of MIC. MLC is the lowest concentration of antimicrobials at which all target cells are dead.

### 4.5. Measurement of Optical Density of Microbial Cultures

The measurement of the optical density (O.D.) is an indication of the number of cells in a liquid culture. In this assay, liquid cultures in TSB or PDB broth were incubated in the presence of 7.5% of each antimicrobial powder, in order to determine the relative ability of each antimicrobial powder to limit microbial growth and thus limit the value of O.D. After sterilization of liquid media (TSB or PDB) and inoculation with 0.1 mL of fresh culture of each target microorganism (bacteria or yeasts), each culture was incubated for 24 h at 37 °C (bacteria) or 25 °C (yeasts). In addition, cultures without any antimicrobial powder were incubated under the same conditions and were used as a control. At the end of the incubation, each culture was diluted at a 1/10 ratio with deionized water and its O.D. was measured in a UV-Vis spectrophotometer (DR 5000, Hach Lange) at 620 nm. The average values of duplicate measurements are reported here.

The target microorganisms used in this assay were: Escherichia coli, Staphylococus aureus, Listeria monocytogenes, Salmonella typhimurium, Bacillus cereus, Campylobacter jejuni, Clostridium perfringens, Saccharomyces cerevisiae, Rhodotorula mucilaginosa, and Candida utilis.

### 4.6. Mycelium Growth Assay

For this method 51 encapsulated powders (Table 1) evaluated against six spoilage fungi, *Penicillium expansum*, *Penicillium italicum* BPIC 1904, *Aspergillus flavus*, *Aspergillus niger*, *Fusarium oxysporum,* and *Geotrichum candidum* for their antifungal activity. From each powder a 10% solution (1 g powder in 10 mL of distilled water) was prepared and then sterilized in an autoclave at 121 °C for 15 min. Then, 20 mL of Potato Dextrose Agar (Oxoid, UK) was poured in petri dishes. Once the petri dishes were cooled down, 0.1 mL of each solution was spread on the plate and left for 1 h so that the solution could be absorbed by the agar. With a sterile glass Paster pipette, a small well was opened in which 25 μL of spore suspension of each fungi was added. An agar plate with each fungi was also prepared without the addition of any antimicrobial substance, which served as a control. All agar plates were incubated at room temperature (25 °C) for 7 days, and the mm of mycelium growth was measured at the end of the incubation period. An average of four measurements was reported as the mean diameter of mycelium expansion for each mold.

### 4.7. In Vivo Antimicrobial Activity (Application in Food Samples)

For the examination of the antimicrobial activity of selected antimicrobial extracts in a realistic food environment, with a concentration of 0.5% the encapsulated powders 100/0/0, 90/10/0, and 80/10/10 were applied and compared to a control treatment without antimicrobials in the following food samples:

(a) A fresh pork/beef burger stored for 4 days at 4 °C;
(b) A fresh cheese spread stored for 21 days at 4 °C;
(c) A fresh yogurt stored for 21 days at 4 °C;
(d) A fresh pasteurized tomato juice stored 21 days at 4 °C.

All food samples were produced in the lab. The burger was prepared from 50% fresh pork and 50% fresh beef minced meat without any other additives or spices. The fresh cheese spread was produced my mixing and homogenizing in a home blender with a mixture of 70% myzithra cheese (a traditional Greek cheese produced from clotted cheese whey) and 30% feta cheese. The fresh yogurt was produced from full fat fresh cow’s milk after a 4 h incubation with yogurt culture at 44 °C, while the tomato juice was produced from sliced and blended fresh tomatoes, which were by condensed by boiling to ¾ of the initial volume (25% condensed) and then pasteurized in glass bottles at 80 °C for 30 min in order to kill any autochthonous fungi present in the raw materials.

The above food samples were either inoculated with pure cultures of pathogens or fungi, or tested for their natural, indigenous microbiota and analyzed in triplicate (the mean values are reported here). In the case of inoculated samples, the microbial cultures were prepared as described in 4.3 and diluted to 1/10 with Maximum Recovery Diluent (Neogen, USA) before being added into the above food samples. All four types of food samples were prepared in triplicate and inoculated with 1% inoculum (1 mL of microbial culture into 100 g of food sample) of food pathogens and spoilage microbiota, as follows: the fresh burgers and fresh cheese spread were inoculated with *Escherichia coli*, *Salmonella typhimurium* and *Listeria monocytogenes*, since these types of food are vulnerable to growth of bacterial pathogens, while the fresh yogurt and tomato juice were inoculated with spoilage fungi, which may occur in such products, namely *Aspergillus niger*, *Penicillium italicum,* and *Rhodotorula mucilaginosa*. The inoculated food samples were homogenized in a stomacher for 1 min in order to distribute the inoculum and they were then stored in sterile containers at 4 °C for 21 days, except for the burgers, which were stored for 4 days. The duration of storage of each sample corresponded to an average ordinary duration of refrigerated storage of fresh burgers, fresh cheese spread, yogurt, and non-sterile or fresh tomato juice.

For the determination of indigenous spoilage indicators, the beef burgers and the pasteurized tomato juice were placed in clean, non-sterile containers in the refrigerator (at 4 °C), after being exposed to natural air contamination and stored for 4 (fresh burger) or 21 (tomato juice) days. The burgers were analyzed for the determination of Total Plate Count (TPC), i.e., the total aerobic mesophilic bacteria, while the total population of yeasts and molds and lactic acid bacteria (LAB) were determined in the tomato juice during storage. These microorganisms were chosen as typical sources of microbial spoilage during storage of fresh minced burgers and opened containers of tomato juice, respectively.

## 5. Conclusions

Using a sustainable, green process for the utilization of olive mill waste, pomegranate, and orange pulp, different combinations of antimicrobial encapsulated extracts were produced and studied in vitro and in food matrices. A concentration of 7.5–10% of the antimicrobial powders could inhibit bacterial growth in vitro, while fungal growth was inhibited at or above 10% content in synthetic growth media. In food samples, the optimal antimicrobial powder containing 100% olive fruit extract led to a reduction of the bacterial population by up to 1.8 log cfu/g, while fungal growth in foods was decreased by up to 2.2 log cfu/g after addition of an encapsulated extract containing 90% olive fruit extract and 10% pomegranate pulp extract. This level of antimicrobial activity is significant, especially in the context of food safety and microbiological quality.

Although processing and extraction conditions may alter the composition and antimicrobial activity of such extracts, the present results show that there is room for potential commercial exploitation of these single or combined fruit extracts in food preservation and in the microbial safety of animals and humans, in an environmentally friendly manner, under the framework of a sustainable and circular economy.

## Figures and Tables

**Table 1:** Concentration of total phenols, flavonoids, and pH values of the selected antimicrobial powders.

Powder Code No.	Phenols g/Kg Powder	Flavonoids g/Kg Powder	pH (of 10% Water Solution)
100/0/0	47.86	51.15	5.08
90/5/5	41.88	54.60	5.07
90/10/0	43.38	59.58	5.10
90/0/10	38.76	55.80	5.05
80/10/10	42.89	57.50	5.03
80/20/0	40.05	65.50	5.07
80/0/20	36.25	51.80	5.01

**Table 2:** Minimum inhibitory concentration of selected antimicrobial powders against several foodborne bacteria and fungi.

Powder.Code No.	*E. coli*	*S. aureus*	*L. monocytogenes*	*S. typhimurium*	*B. cereus*	*C. jejuni*	*C. perfingens*	*P. expansum*	*A. niger*	*A. flavus*	*P. italicum*	*F. oxysporum*
100/0/0	10%	10%	7.5%	7.5%	7.5%	7.5%	10%	>10%	>10%	>10%	>10%	7.5%
90/5/5	10%	7.5%	7.5%	7.5%	7.5%	7.5%	10%	>10%	>10%	>10%	>10%	7.5%
90/10/0	7.5%	7.5%	7.5%	7.5%	7.5%	7.5%	10%	>10%	>10%	>10%	>10%	10%
90/0/10	10%	10%	7.5%	7.5%	7.5%	10%	10%	>10%	>10%	>10%	>10%	10%
80/10/10	10%	7.5%	7.5%	7.5%	7.5%	7.5%	10%	>10%	>10%	>10%	>10%	7.5%
80/20/0	10%	7.5%	7.5%	7.5%	7.5%	7.5%	10%	>10%	>10%	>10%	>10%	10%
80/0/20	10%	>10%	7.5%	7.5%	7.5%	10%	10%	>10%	>10%	>10%	>10%	10%

**Table 3:** Coding of samples of selected antimicrobial powders and relative ratio (%) of the three different extracts of olive, pomegranate, and orange.

Powder Code No.	Olive Extract Ratio	Pomegranate Extract Ratio	Orange Extract Ratio
100/0/0	100	0	0
90/5/5	90	5	5
90/10/0	90	10	0
90/0/10	90	0	10
80/10/10	80	10	10
80/20/0	80	20	0
80/0/20	80	0	20

## Data Availability

The data presented in this study are available on request from the corresponding author.

## Sample Availability

Samples of the compounds are available from the authors.
